# 

**Published:** 1876-02

**Authors:** 


### Contents of February Number.

### ORIGINAL.

ART. I.—On the use of Warm and Hot Water in Surgery. By Frederick E. Hyde, M. D., ........ 241

ART. II.—Certain Points connected with Typhoid Fever. By F. C. Curtis, M. D., ........ 255

ART. III.—Medical Society of the County of Albany. Semi-Monthly Meeting, January 5th, 1876, ........ 267

Correspondence, ........ 272

On the use of Actual Cautery in the Enucleation of Fibroids of the Uterus, 273

### EDITORIAL DEPARTMENT.

Meeting of the Alumni Association of the Buffalo Medical College, ........ 274

BOOKS REVIEWED:

- Cyclopædia of the Practice of Medicine, Vol V, ........ 277
- Lectures on Syphilis. By Henry Lee, M. D., ........ 278
- A Text Book of Human Physiology. By Austin Flint, Jr., M. D. 279
- On Poisons in Relation to Medical Jurisprudence. By Alfred S. Taylor, M. D., F. R. S., ........ 279

Books and Pamphlets Received, ........ 280

